# Biosynthesis of Silver Nanoparticles Using *Azadirachta indica* and Their Antioxidant and Anticancer Effects in Cell Lines

**DOI:** 10.3390/mi13091416

**Published:** 2022-08-28

**Authors:** S. Anitha Kumari, Anita K. Patlolla, P. Madhusudhanachary

**Affiliations:** 1 Department of Zoology, Osmania University for Women, Hyderabad 500095, India; 2 RCMI Center for Environmental Health, College of Science, Engineering and Technology, Jackson State University, Jackson, MS 39217, USA; 3 Ultrastructure Unit, National Institute of Nutrition, Hyderabad-500095, India

**Keywords:** *Azadirachta indica*, green synthesis, silver nanoparticles, DPPH, MTT

## Abstract

In the present study, silver nanoparticles (Ag-NPs) were synthesized using *Azadirachta indica* extract and evaluated for their in vitro antioxidant activity and cytotoxicity efficacy against MCF-7 and HeLa cells. The silver nanoparticles (Ag-NPs) were formed within 40 min and after preliminary confirmation by UV-visible spectroscopy (peak observed at 375 nm), they were characterized using a transmission electron microscope (TEM) and dynamic light scattering (DLS). The TEM images showed the spherical shape of the biosynthesized Ag-NPs with particle sizes in the range of 10 to 60 nm, and compositional analysis was carried out. The cytotoxicity and antioxidant activity of various concentrations of biosynthesized silver nanoparticles, *Azadirachta indica* extract, and a standard ranging from 0.2 to 1.0 mg/mL were evaluated. The 2,2-Diphenyl-1-picrylhydrazyl (DPPH) activity of the biosynthesized Ag-NPs and aqueous leaf extract increased in a dose-dependent manner, with average IC_50_ values of the biosynthesized Ag-NPs, aqueous leaf extract, and ascorbic acid (standard) of 0.70 ± 0.07, 1.63 ± 0.09, and 0.25 ± 0.09 mg/mL, respectively. Furthermore, higher cytotoxicity was exhibited in both the MCF-7 and HeLa cell lines in a dose-dependent manner. The average IC_50_ values of the biosynthesized Ag-NPs, aqueous leaf extract, and cisplatin (standard) were 0.90 ± 0.07, 1.85 ± 0.01, and 0.56 ± 0.08 mg/mL, respectively, with MCF-7 cell lines and 0.85 ± 0.01, 1.76 ± 0.08, 0.45 ± 0.10 mg/mL, respectively, with HeLa cell lines. Hence, this study resulted in an efficient green reductant for producing silver nanoparticles that possess cytotoxicity and antioxidant activity against MCF-7 and HeLa cells.

## 1. Introduction

Silver nanoparticles are among the most vital and fascinating nanomaterials and are involved in the production of multiple consumer products such as textiles, cosmetics, bandages, cleaning products, and contraceptives, as well as life sciences and biomedical applications [1,2]. Ag-NPs have been reported to possess several functional activities, such as antibacterial, antifungal, antiviral, anti-inflammatory, antiangiogenic, antioxidant, and anticancer properties, and they can effectively kill a variety of pathogens even at very low concentrations compared to standard antimicrobial agents [3]. Ag-NPs are prevalent in disease management because of their specific interaction and disruption of mitochondrial function by inducing the generation of reactive oxygen species and suppressing ATP synthesis, which finally leads to DNA damage [4].

Various techniques are available for the synthesis of Ag-NPs with controlled size and shape, and specific synthetic methods have been established including physical, chemical, and biological methods [5]. Physical synthesis can obtain Ag-NPs with uniform size distribution and high purity [6]. Chemical synthesis is the most-used procedure to acquire Ag-NPs [6] involving the reduction of silver ions to silver atoms [7]. Besides reducing agents, capping agents and stabilizers are also involved in obtaining Ag-NPs with suitable properties. Although chemical methods of Ag-NP synthesis are universally used, the toxicity and pollution caused by the chemicals may pose potential environmental and biological risks; thus, there is a growing need for green synthesis that includes clean, non-toxic, and environmentally friendly methods of synthesizing nanoparticles with sustainable commercial viability [8]. Recently, green nanotechnologies have become increasingly popular and have significant applications in various biomedical fields. Techniques for obtaining nanoparticles using naturally occurring reagents such as sugars, microorganisms, fungi, enzymes, plants, or plant extracts as reductants and capping agents are used as viable alternatives to chemical and physical methods [9].

For many decades, the incidence of breast cancer and cervical cancer among women in developing countries has increased worldwide, and efforts to fight against these deadly diseases are growing [10,11]. Among the various cell lines used for the study of breast cancer, MCF-7 is the most studied [12]. Further, immortalized human cancer cell lines, such as HeLa, constitute a crucial scientific tool for the study of cervical cancer [13]. Several therapeutic approaches for the treatment of cancer have been developed using Ag-NPs. Because of their wide applications, many methods have been developed for the synthesis of Ag-NPs that are capable of controlling their size. Therefore, Ag-NPs with small particle size and no bulking between the particles are highly favorable [14]. Several physical, chemical, and biological methods are known for the preparation of metal nanoparticles [15]. The physical and chemical methods are highly costly and toxic to the environment [16].

Although the potential of higher plants as a source for the green synthesis of nanoparticles is still largely unexplored, plants provide better options for nanoparticle synthesis compared to other biological molecules because they are mostly non-toxic, provide natural capping agents, and eliminate the elaborate procedures of maintaining microbial cultures [17]. Different plant extracts including *Azadirachta indica*, *Z. officinale* (ginger), *C. frutescens* (cayenne pepper), *A. sativum* (garlic) [18], marigold flower [19], *Ziziphora tenuor* [20], and *Ocimum tenuiflorum* [21] have been successfully reported for the biosynthesis of Ag-NPs as alternatives to conventional methods. *Azadirachta indica* (neem) is a medicinal plant that belongs to the Meliaceae family and has well-established antimicrobial properties [22]. The antibiotic properties of *Azadirachta indica* leaf extract are due to its constituent organic compounds [23]. The leaf extract of *Azadirachta indica* is a storehouse of many phytochemicals [24], some of which act as reducing agents for AgNO_3_, leading to the synthesis of Ag-NPs. The antimicrobial activity of Ag-NPs synthesized using *Azadirachta indica* has been widely reported [25,26,27,28]. Recent studies reported on Ag-NPs synthesized using the leaf extract of *Azadirachta indica* and are being utilized in the development of wash-durable antimicrobial cotton fabrics [29] and in the degradation of textile effluents [30]. However, the effect of the *Azadirachta indica* leaf extract-mediated synthesis of Ag-NPs on anticancer and antioxidant activities has not been reported. Therefore, the aim of this study was to evaluate the antioxidant and anticancer properties of biosynthesized Ag-NPs on MCF-7 and HeLa cells, suggesting their potential therapeutic use in the treatment of cancer.

## 2. Materials and Methods

### 2.1. Chemicals

Silver nitrate was purchased from Merck (Mumbai, India), along with Dulbecco’s modified Eagle’s medium (DMEM), MTT (3-(4,5-dimethylthiazol-2-yl)-2,5-diphenyl tetrazolium bromide), and trypsin. EDTA and phosphate-buffered saline (PBS) were purchased from Sigma-Aldrich (St. Louis, MO, USA), and fetal calf serum (FBS) was purchased from Gibco. Whatman filter paper no. 42 25 cm^2^, a 75 cm^2^ flask, and 96-well plates were purchased from Eppendorf, India.

### 2.2. Experimental Design

Typically, plant extract-mediated bio-reduction involves mixing the aqueous extract with an aqueous solution of the appropriate metal salt. The synthesis of nanoparticles occurs at room temperature and is completed within a few minutes.

### 2.3. Preparation of Plant Extract

*A. indica* (neem) leaf extract was used to prepare silver nanoparticles based on the cost effectiveness, ease of availability, and medicinal properties. Fresh leaves were collected from a greenhouse facility in Hyderabad (Telangana, Hyderabad, India). The surfaces of the leaves were cleaned with running tap water to remove debris and other contaminated organic contents, followed by washing with deionized water and air drying at room temperature. About 15 gm of finely cut leaves was kept in a 250 mL Erlenmeyer flask containing 50 mL deionized water and boiled for 25 min at 60 °C in a water bath. The extract was cooled down to room temperature, filtered with Whatman filter paper no. 42 under vacuum, and stored at 4 °C for further use.

### 2.4. Green Synthesis of Silver Nanoparticles

**Figure 1:**
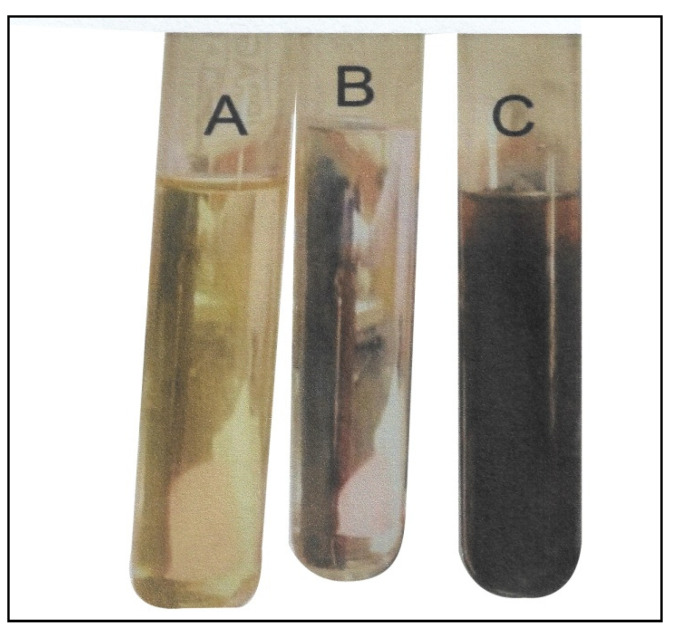
Visual observation of silver nanoparticle (Ag-NP) formation (**A**) *Azadirachta indica* extract; (**B**) silver nitrate solution and (**C**) silver nanoparticles.

A volume of 100 mL of 1 mM solution of silver nitrate (AgNO_3_) was prepared in an Erlenmeyer flask. The Ag-NPs were synthesized by adding 10 mL of *A. indica* (neem) leaf extract to 50 mL of 1 mM aqueous AgNO_3_ solution at room temperature, stirring continuously for 20 min. The mixture obtained was incubated in a dark chamber at room temperature to prevent the auto-oxidation of the silver nitrate. The reduction of silver ions to silver nanoparticles was confirmed by the color change of the solution from reddish to dark brown (Figure 1), indicating the synthesis of Ag-NPs, and the formation was also confirmed using UV-visible spectroscopy.

### 2.5. Characterization of the Biosynthesized Silver Nanoparticle (Ag-NP)

The solution containing Ag-NPs was centrifuged at 2000 rpm after 24 h for 15 min, and the resulting pellets were dried in an oven at 100 °C for 24 h. The purified biosynthesized Ag-NPs were characterized using the following techniques.

### 2.6. UV-Vis Spectroscopy

**Figure 2:**
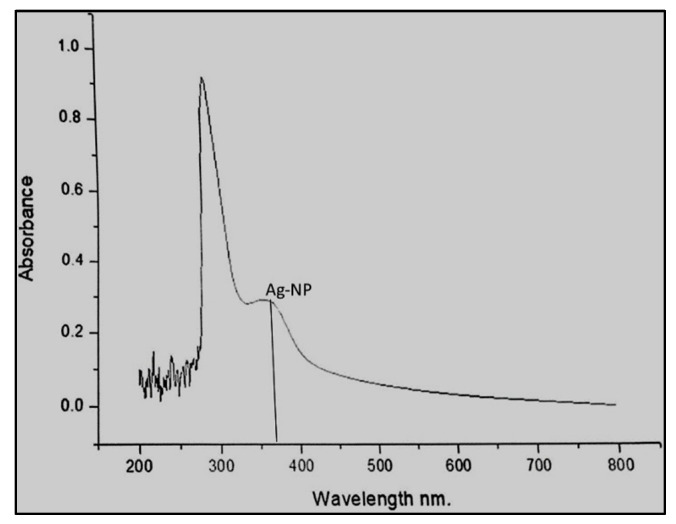
UV-visible spectra of silver nanoparticles biosynthesized after 48 h of incubation.

The UV-visible spectrophotometric analysis of the synthesized Ag-NPs solutions was carried out at room temperature using a V-670 UV-Vis spectrophotometer (JASCO) with a resolution of 0.5 nm. The absorbance of the sample was read at the wavelengths of the 200 to 700 nm range. One milliliter of the sample was pipetted into a test tube and subsequently analyzed at room temperature (Figure 2).

The chemical structure of the biosynthesized Ag-NPs samples was determined using transmission electron microscopy and dynamic light scattering (DLS).

### 2.7. TEM Analysis

**Figure 3:**
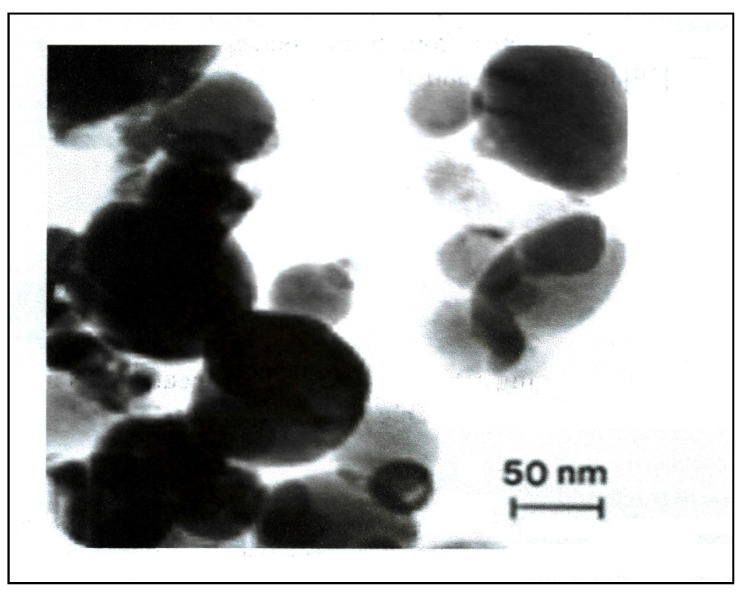
TEM images of biosynthesized silver nanoparticles.

The size, shape, and morphology of the biosynthesized silver nanoparticles were determined using a transmission electron microscope (TEM). The samples for the TEM were prepared by sonicating the pellet of centrifuged Ag-NPs in deionized water. A drip of the homogeneous suspension was placed on a carbon-coated copper grid with a lacey carbon film and allowed to dry at room temperature. The images (Figure 3) were collected using a field emission JEOL-JEM-2100F TEM operating at 200 KV (JEOL, Tokyo, Japan).

### 2.8. Dynamic Light Scattering (DLS)

**Figure 4:**
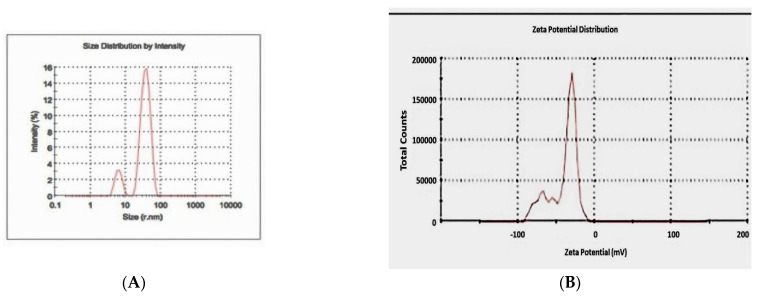
Particle size distribution of the biosynthesized silver nanoparticles by DLS (**A**) and zeta potential (**B**).

The nanostructure size and zeta potential were measured in deionized water (DI) using a Zetasizer (Malvern, Worcestershire, UK). The biosynthesized Ag-NPs were briefly measured after the dilution of a stock solution of 50 µg/mL in sterile water. These dilutions were vortexed and sonicated for 5 min to provide a homogeneous dispersion. Then, 1 mL of the diluted dispersion of the Ag-NPs was transferred to a 1 cm^2^ cuvette for dynamic size measurement (Figure 4A). To measure the zeta potential, a Malvern zeta potential cell was washed 3–5 times with ultrapure water followed by transferring 850 µL of diluted dispersion of biosynthesized Ag-NPs to this cell to measure the zeta potential (Figure 4B). Following this, 60 nm NIST standard gold nanoparticles were used in the validation of the instrument. Both the size and zeta potential were measured at least three times. The data were calculated as the average size or zeta potential of the Ag-NPs.

### 2.9. Cell Culture

MCF-7 and HeLa cell lines were procured from the National Centre for Cell Science (NCCS), Pune (India), and were thawed by gentle agitation of their containers (vials) for 2 min in a water bath at 37 °C. After thawing, the content of each vial was transferred to a 75 cm^2^ tissue culture flask, diluted with the DMEM supplemented with 10% FBS and 1% penicillin and streptomycin, and incubated for 2 to 3 days at 37 °C in a 5% CO_2_ incubator. The growth medium was changed twice a week. Cells grown to 75–85% confluence were washed with phosphate buffer saline (PBS), trypsinized with 3 mL of 0.25% (v) trypsin—0.3%/v EDTA, diluted with fresh medium, and counted using a hemacytometer.

### 2.10. DPPH (2,2-Diphenyl-1-Picrylhydrazyl) Assay

The antioxidant activity of the biosynthesized Ag-NPs was determined using the DPPH method [31] with slight modifications. The DPPH assay is based on the ability of DPPH, a stable free radical, to decolorize in the presence of an antioxidant. It is a direct and reliable method for determining the radical scavenging action of a chemical.

Five different concentrations of biosynthesized Ag-NP ranging from 0.2, 0.4, 0.6, 0.8, and 1.0 mg/mL was placed in individual cuvettes, and about 3 mL of 0.1 mM methanolic solution of DPPH radical was added. The mixture was vigorously shaken and allowed to stand for 30 min in the dark at room temperature. The control contained all of the reaction reagents except the biosynthesized Ag-NP, and methanol was used for baseline correction. The absorbance was then measured at 517 nm using a spectrophotometer. The results were compared with the standard oxidants. The ability of DPPH scavenging activity was then calculated using the formula

DPPH scavenging activity (% of Inhibition) = AO − A1/AO × 100

where AO is the absorbance of the control and A1 is the absorbance of the test sample (biosynthesized Ag-NPs).

The results of the antioxidant activity are expressed as IC_50_. The IC_50_ is the concentration (µg) of the test sample required to scavenge 50% of the radicals or inhibit 50% of DPPH concentration. The IC_50_ value was calculated using linear regression analysis.

### 2.11. MTT Assay

The anticancer activity of the biosynthesized Ag-NPs was assessed using the MTT assay [32] in human MCF-7 and HeLa cells. The assay is based on the reduction of MTT (3-(4,5-dimethyl)-2-thiazolyl)-2,5-diphenyl-2 H tetrazolium bromide) by mitochondrial dehydrogenase to purple formazan. To conduct this assay, 180 µL aliquots in six replicates of the cell suspension (5 × 10^5^/mL) were seeded to 96-well polystyrene tissue culture plates, and 20 µL aliquots of stock solutions were added to each well using deionized water as a solvent to make up the final biosynthesized Ag-NP doses of 0.2, 0.4, 0.6, 0.8, and 1.0 mg/mL. The control cells received 20 µL of deionized water. All chemical exposures were carried out in 96-well tissue culture plates for the purpose of chemical dilutions. The cells were placed in a humidified 5% CO_2_ incubator for 48 h at 37 °C. After incubation, 20 µL aliquots of MTT solution (5 mg/mL in PBS) were added to each well and re-incubated for 4 h at 37 °C followed by low centrifugation at 800 rpm for 5 min. Then, 200 µL of supernatant culture medium was carefully aspirated, and 200 µL aliquots of dimethyl sulfoxide (DMSO) were added to each well to dissolve the formazan crystals, followed by incubation for 10 min to dissolve any air bubbles. The culture plate was placed on a Biotek microplate reader, and the absorbance was measured at 590 nm.

Cell viability = OD of sample − OD of control/OD of control × 100.
(OD = optical density)

### 2.12. Statistical Analysis

Multiple linear regression analysis was used for the comparison of the data through Statistica version 5.0 (Statsoft, Hyderabad, India). The results are expressed as the mean ± SD The value of *p* < 0.05 was considered statistically significant.

## 3. Results and Discussion

Visual Observation and UV-Visible Spectroscopy

The aqueous extract of *Azadirachta indica* was able to bio-reduce the AgNO_3_ to Ag-NPs. The biosynthesized silver nanoparticle was characterized, and its anticancer and antioxidant activities were studied in MCF-7 and HeLa cell lines.

The silver nanoparticle formation was assessed visually by color change. The addition of aqueous *Azadirachta indica* extract (neem) to the AgNO_3_ solution resulted in a color change of the mixture from yellowish to reddish brown, indicating Ag-NP formation (Figure 1). The color change observed in the formation of the biosynthesized silver nanoparticles was due to the surface plasmon resonance (SPR) of the nanoparticles in the reaction mixture. This was also reported by several other studies that observed a color change in the reaction mixture during the formation of silver nanoparticles biosynthesized from *Oscillatoria species, Allium sativum, Zingiber officinale*, *and Capsicum frutescens* [18,33].

The biosynthesized Ag-NPs were characterized 48 h after the incubation using UV-visible spectroscopy. The surface plasmon resonance (SPR) peak was observed at 375 nm, and the broad-spectrum range was 200–500 nm (Figure 2). The results obtained from the UV-Vis spectra indicate that silver nanoparticles were formed. In our studies, the plasmon resonance band of the biosynthesized Ag-NPs was observed at 375 nm compared to certain other reports, where the bands were mostly shown in the range of 435 to 445 nm. This effect may be due to the concentration of the leaf extract being low in our sample, and such effects have been reported by Ahmed et al. [17] where increasing the concentration of the plant extract was shown to increase the intensity of absorption. However, there is one study by Otunola et al. [18] where the UV-Vis absorption of Ag-NPs for garlic extract was 375 nm. The lower plasmon resonance band in our study compared to other reports could also be due to the incubation time.

Transmission electron microscopy was used to identify the size, shape, and morphology of the nanoparticles. It showed that silver nanoparticles are well dispersed and predominantly spherical in shape, as shown in Figure 3. The nanoparticles are homogeneous and spherical, which conforms to the shape of SPR band in the UV-visible spectrum. The results from DLS showed the agglomeration of the biosynthesized Ag-NPs at more than their primary size, and the zeta potential value was shown to be −33.2 mV, revealing that the synthesized Ag-NPs are highly stable (Figure 4). The particle size agrees with that calculated from DLS with an average diameter of 38.5 nm (86.4%) and at least 6.5 nm diameter (13.6%).

**Figure 5:**
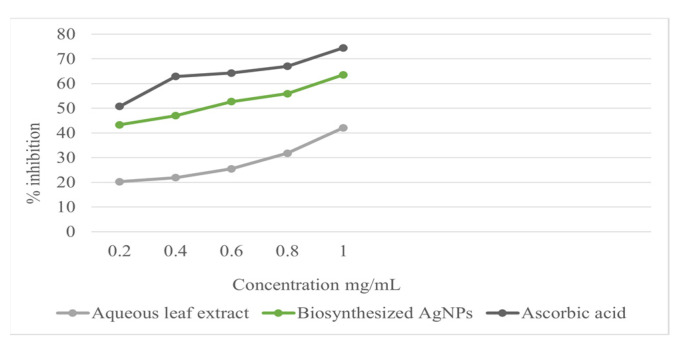
DPPH radical scavenging activity of different concentrations of aqueous leaf extract, biosynthesized Ag NPs, and ascorbic acid.

Using the stable radical DPPH assay, the antioxidant activity of the Ag-NPs was measured in terms of hydrogen donating or radical scavenging ability. The effect of the different concentrations of the biosynthesized Ag-NPs and the aqueous leaf extract on DPPH radical scavenging activity is shown in Table 1. The biosynthesized Ag-NPs exhibited more scavenging activity of DPPH than the aqueous leaf extract. The DPPH activity of biosynthesized Ag-NPs and the aqueous leaf extract was found to increase in a dose-dependent manner, with average IC_50_ values of biosynthesized Ag-NPs, aqueous leaf extract, and ascorbic acid (standard) of 0.70 ± 0.07, 1.63 ± 0.09, and 0.25 ± 0.09 mg/mL, respectively. The biosynthesized Ag-NPs thus exhibited a dose-dependent increase with a lower DPPH radical scavenging ability. Ag-NPs produced DPPH scavenging power comparable to ascorbic acid (Figure 5 and Table 1). The DPPH scavenging activity in this study indicated that biosynthesized Ag-NPs are a potent antioxidant and are further capable of donating a hydrogen to a free radical in order to remove the odd electron, which is responsible for the free-radical reactivity. The antioxidant activities were enhanced by conversion into Ag-NPs. Polyphenolic compounds in plants have been reported to have strong antioxidant properties, which help to protect cells against oxidative stress by free radicals. Similar studies of the enhanced antioxidant properties of biosynthesized Ag-NPs from plant sources such as Piper *longum* [34] and Chenopodium *murale* [35] have been reported.

The data are presented as mean ± SD (*p* < 0.05). IC_50_ is the concentration of aqueous leaf extract and biosynthesized silver nanoparticles (Ag-NPs) causing 50% DPPH radical scavenging activity.

All parts of the neem tree and its constituents have been demonstrated to exhibit a wide range of medicinal properties, especially antioxidant and anticancer properties. Neem leaf aqueous extract effectively suppresses different types of cell carcinomas [36]. Further, researchers have shown prominent anticancer activities from limonoid-derived compounds from neem. A very recent study showed that neem leaf ethanolic extracts stopped cell growth and induced apoptosis in both the estrogen-independent MDAMB-231 and estrogen-dependent MCF-7 cell lines of breast cancer in humans [37]. Therefore, in this study, we performed an MTT [32] assay to evaluate the anticancer activity of the biosynthesized Ag-NPs.

The anticancer activity of different concentrations of biosynthesized Ag-NPs and the aqueous leaf (neem) extract in MCF-7 and HeLa cell lines according to the MTT assay are shown in Table 2. The biosynthesized Ag-NPs and the aqueous leaf extract exhibited higher cytotoxic activity in both MCF-7 and HeLa cell lines in a dose-dependent manner (Table 2). The average IC_50_ values of the biosynthesized Ag-NPS, aqueous leaf extract, and cisplatin (standard) were 0.90 ± 0.07, 1.85 ± 0.01, and 0.56 ± 0.08 mg/mL, respectively, for MCF-7 cell lines and 0.85 ± 0.01, 1.76 ± 0.08, and 0.45 ± 0.10 mg/mL, respectively, for HeLa cell lines. The biosynthesized Ag-NPs showed a higher percentage of cytotoxicity compared to the aqueous leaf extract. These results indicate that the Ag-NPs significantly decreased the growth of both MCF-7 and HeLa cell lines. Several studies [38,39,40] on the anticancer activity of biosynthesized Ag-NPs in vitro have been reported and show similar results to our outcome.

The data are presented as mean ± SD (*p* < 0.05). The IC_50_ concentration of aqueous leaf extract and biosynthesized silver nanoparticles (Ag-NPs) caused 50% cytotoxic activity.

Lower IC_50_ values indicate greater antioxidant activity [41]. In the present study, it was found that the incubation of both cancer cell lines, MCF-7 and HeLa, with biosynthesized Ag-NPs reduced the viability of these cells, and the dead cells were significantly increased with a higher concentration of biosynthesized Ag-NPs. Even at a very low concentrations, Ag-NPs showed 20% dead cells. Fard et al. [42] reported similar results in which Ag-NPs synthesized using Artemisia oliveriana extract showed apoptosis in A549 cells after the treatment. Several studies [43,44,45] demonstrated reduced cell viability after exposure to biosynthesized Ag-NPs. The findings of the present study indicate that biosynthesized Ag-NPs have strong antioxidant and anticancer properties. Hence, this study resulted in an efficient green reductant for producing silver nanoparticles that possess cytotoxicity and antioxidant activity against MCF-7 and HeLa cells.

## 4. Conclusions

Ag-NPs were successfully synthesized from AgNO_3_ and aqueous extract of *Azadirachta indica* (neem), thus revealing an excellent application of green synthesis. The biosynthesized Ag-NPs exhibited strong antioxidant and anticancer activity in MCF-7 and HeLa cells. Further optimization of the current green synthesis method would help in the production of monodispersed Ag-NPs, suggesting its potential therapeutic use in cancer and other diseases as they are non-toxic, cost effective, and environmentally safe.

## Figures and Tables

**Table 1:** DPPH radical scavenging activity of *Azadirachta indica* (neem), aqueous leaf extract, biosynthesized silver nanoparticles (Ag-NPs), and ascorbic acid (standard).

Sample	Concentration in mg/mL	Scavenging Activity in %	IC_50_ Value in mg/mL
Aqueous leaf extract	0.2 0.4 0.6 0.8 1.0	20.28 ± 0.79 21.89 ± 0.64 25.49 ± 1.95 31.75 ± 1.81 42.11 ± 2.03	1.63 ± 0.09
Biosynthesized silver nanoparticles (Ag-NPs)	0.2 0.4 0.6 0.8 1.0	43.29 ± 2.16 46.98 ± 2.12 52.68 ± 3.89 55.89 ± 2.25 63.57 ± 2.41	0.70 ± 0.07
Ascorbic acid (standard)	0.2 0.4 0.6 0.8 1.0	50.78 ± 1.09 62.89 ± 0.60 64.28 ± 0.30 66.99 ± 0.32 74.48 ± 1.20	0.25 ± 0.09

**Table 2:** Anticancer activity of *Azadirachta indica* (neem), aqueous leaf extract, biosynthesized silver nanoparticles (Ag-NPs), and cisplatin (standard) in MCF-7 and HeLa cell lines.

Sample	Concentration in mg/mL	% of Cytotoxicity MCF-7 Cell Lines	IC_50_ Value in mg/mL MCF-7 Cell Lines	% of Cytotoxicity HeLa Cell Lines	IC_50_ Value in mg/mL HeLa Cell Lines
Aqueous leaf extract	0.2 0.4 0.6 0.8 1.0	21.93 ± 1.95 23.61 ± 2.22 26.68 ± 1.95 32.64 ± 0.74 43.94 ± 1.81	1.85 ± 0.01	20.64 ± 0.81 22.42 ± 1.62 27.01 ± 1.95 31.54 ± 0.89 44.20 ± 1.20	1.76 ± 0.08
Biosynthesized silver nanoparticles (Ag-NPs)	0.2 0.4 0.6 0.8 1.0	45.93 ± 3.22 47.78 ± 2.66 52.68 ± 1.95 58.27 ± 2.01 65.78 ± 0.74	0.90 ± 0.07	43.20 ± 1.78 46.19 ± 2.13 51.40 ± 1.80 55.89 ± 2.26 63.20 ± 2.20	0.85 ± 0.01
Cisplatin (standard)	0.2 0.4 0.6 0.8 1.0	51.53 ± 0.95 61.13 ± 1.66 63.17 ± 2.60 65.42 ± 2.01 70.50 ± 1.99	0.56 ± 0.08	50.64 ± 1.08 62.89 ± 0.70 64.19 ± 0.40 65.20 ± 0.14 71.53 ± 0.95	0.45 ± 0.10
